# Heparanase is a prognostic biomarker independent of tumor purity and hypoxia based on bioinformatics and immunohistochemistry analysis of esophageal squamous cell carcinoma

**DOI:** 10.1186/s12957-022-02698-9

**Published:** 2022-07-16

**Authors:** Yu Wang, Tongjun Song, Kai Li, Hao Liu, Yan Han, Tao Xu, Fengjun Cao, Yong Li, Yuandong Yu

**Affiliations:** 1 Department of Oncology, Renmin Hospital, Hubei University of Medicine, Shiyan, Hubei 442000 People’s Republic of China; 2 Department of Pathology, Renmin Hospital, Hubei University of Medicine, Shiyan, Hubei 442000 People’s Republic of China

**Keywords:** Tumor purity, Hypoxia, Esophageal squamous cancer, Immunohistochemical staining

## Abstract

**Background:**

Esophageal squamous cell carcinoma (ESCC) is a common malignant tumor of the digestive tract with a poor prognosis. The tumor microenvironment (TME) is mainly composed of tumor cells, stromal cells, and immune cells and plays an important role in ESCC development. There are substantial differences in tumor purity among different parts of ESCC tissues, consisting of distinct immune and stromal cells and variations in the status of hypoxia. Thus, prognostic models of ESCC based on bioinformatic analysis of tumor tissues are unreliable.

**Method:**

Differentially expressed genes (DEGs) independent of tumor purity and hypoxia were screened by Spearman correlation analysis of public ESCC cohorts. Subsequently, the DEGs were subjected to Cox regression analysis. Then, we constructed a protein–protein interaction (PPI) network of the DEGs using Cytoscape. Intersection analysis of the univariate Cox and PPI results indicated that heparanase (HPSE), an endo-β-D-glucuronidase capable of cleaving heparan sulfate side chains, was a predictive factor. Gene set enrichment analysis (GSEA) was used to reveal the potential function of HPSE, and single-cell sequencing data were analyzed to evaluate the distribution of HPSE in immune cells. Furthermore, a human ESCC tissue microarray was used to validate the expression and prognostic value of HPSE.

**Result:**

We found that HPSE was downregulated in ESCC tissues and was not correlated with tumor purity or hypoxia status. HPSE is involved in multiple biological processes. ESCC patients with low HPSE expression in cancerous tissues exhibited poor prognosis.

**Conclusions:**

These results indicate that low HPSE expression in cancerous tissues correlates with poor prognosis in patients with ESCC. HPSE is a novel prognostic biomarker independent of tumor purity and hypoxia status in ESCC.

**Supplementary Information:**

The online version contains supplementary material available at 10.1186/s12957-022-02698-9.

## Background

Esophageal carcinoma is one of the most common malignant tumors and has high morbidity and mortality rates in China and worldwide [1]. According to histological classification, esophageal carcinoma is divided into esophageal squamous cell carcinoma (ESCC) and esophageal adenocarcinoma (EAC) [2]. ESCC is the predominant histopathologic subtype and accounts for approximately 90% of esophageal cancer cases in China [3]. Despite recent advances in esophageal carcinoma treatment, the prognosis of ESCC remains poor. Several genes have been reported as prognostic factors in patients with esophageal carcinoma [4]. However, these genes are not sufficient for the clinical diagnosis and prognostic evaluation of esophageal carcinoma.

In recent years, the tumor microenvironment (TME) has been revealed to play an important role in tumor development. The TME is mainly composed of tumor cells, stromal cells, and immune cells [5, 6]. Because different tumor tissues contain distinct immune cells and stromal cells, there are substantial differences in tumor purity in different parts of tumor tissues. Thus, gene expression analysis based on tumor tissue is unreliable. There is still a challenge in performing precise genetic analysis that indicates the dynamic modulation of the immune and stromal components in the TME.

Hypoxia is a typical characteristic of the TME, which is markedly different from the environment of normal tissues [7, 8]. The TME can become hypoxic. Previous studies have shown that the degree of tumor hypoxia is closely associated with tumor development, proliferation, metastasis, and treatment responses [9]. There is, however, a clear difference in gene expression profiles and tumor hypoxia statuses in different parts of tumor tissues.

Given these differences in tumor purity and hypoxia status, the screening of prognostic biomarkers based on gene expression in tumor tissues is unreliable. In the present study, we identified a novel prognostic biomarker independent of tumor purity and hypoxia based on bioinformatics analysis in ESCC. We found that the expression of HPSE was downregulated and not influenced by tumor purity or hypoxia in ESCC tissues. HPSE might be a potential prognostic indicator in ESCC. Our study will be helpful in the diagnosis of ESCC and has great clinical value.

## Materials and methods

### Data processing and the identification of DEGs

Microarray data were downloaded from the Gene Expression Omnibus (GEO) database (http://www.ncbi.nlm.nih.gov/geo/). The GSE44021 [10], GSE67269 [10], GSE38129 [11], and GSE53625 [12] datasets were downloaded for our study. GSE53625 contained 179 paired ESCC and noncancerous tissues with clinicopathological parameters. GSE44021, GSE67269, and GSE38129 contained 287 paired ESCC and adjacent normal tissues. These four gene sets were used to analyze the differentially expressed genes between tumoral and nontumoral tissues. The “limma” R package was used to identify the differentially expressed genes (DEGs) between tumor tissues and adjacent nontumorous tissues. Adjusted *p* value < 0.05 and |log2| fold change (FC)| > 1 were regarded as the screening conditions for DEGs.

### Analysis of the correlation between tumor purity and gene expression

To evaluate the tumor stromal and immune signatures, we applied the R package “estimate” to calculate the immune/stromal scores of the ESCC samples for tumor purity prediction. The correlations between tumor purity and gene expression levels were analyzed by Spearman’s test. |Spearman correlation coefficient| ≥ 0.4 and a *p* value ≤ 0.001 were considered to indicate significance.

### Definition of hypoxia-related genes

To determine the effects of hypoxia, we collected hypoxia-related genes from the Molecular Signatures Database V7.2 (https://www.gsea-msigdb.org/gsea/msigdb, Hypoxia M10508). The hypoxia gene sets were scored with the gene set variation analysis (GSVA) method. We then performed a coexpression analysis of the DEGs and hypoxia-related genes in each gene set based on the Spearman correlation analysis. The genes with a |Spearman correlation coefficient| ≥ 0.4 and a *p* value ≤ 0.001 in each gene set were confirmed as hypoxia-related genes.

### Gene ontology (GO) and Kyoto encyclopedia of genes and genomes (KEGG) enrichment analysis and gene set enrichment analysis

GO and KEGG enrichment analyses were used to identify the biological functions and related regulatory pathways of the candidate gene set. The “clusterProfiler” R package was used for GO and KEGG enrichment analyses of the DEGs, and terms with *p* value of <0.05 were considered significantly enriched. Gene set enrichment analysis (GSEA) (version 4.0.1, http://www.broadinstitute.org/gsea) was also performed to assess the differences in the enriched gene sets between the low- and high-risk groups. Gene set permutations were performed 1000 times for each analysis. The whole transcriptome of all tumor samples was used for GSEA, and only gene sets with NOM *p* < 0.05 were considered significant. The Reactome database was used to explore the steps of HPSE biological pathways.

### PPI network construction and cox regression analysis

PPI network establishment Search Tool for the Retrieval of Interacting Genes/Proteins (STRING) (https://www.string-db.org/) [13] was designed to evaluate protein–protein interaction (PPI) network information. Then, we used Cytoscape software (version 3.6.1) for PPI network visualization, and nodes with interaction confidence values larger than 0.90 were used for building the network. Furthermore, the survival package in R software was used to construct a Cox regression model.

### Single-cell sequencing data analysis

Single-cell sequencing data were obtained from dataset GSE160269 in the GEO database. Bioinformatics data were processed using Seurat (version 3.1.2). The Seurat package was used for quality control, and the remaining 70,556 cells were normalized. Principal component analysis (PCA) was completed for preliminary dimension reduction. Unsupervised clustering analysis using Seurat identified 8 distinct immune cell clusters. The Uniform manifold approximation and projection (UMAP) technique was employed for visualization. Expression levels of HPSE marker genes across 70,556 cells were shown as UMAP plots.

### Tissue microarray and immunohistochemistry (IHC) analysis

All paraffin-embedded ESCC specimens were obtained from the Biobank of the National Engineering Center for Biochip in Shanghai. All detected specimens were derived from ESCC tissues acquired via surgical resection or biopsy. This study was approved by the Ethics Committees of National Engineering Center for Biochip in Shanghai (the National Human Genetic Resources Sharing Service Platform, No. 2005DKA21300). Informed consent was obtained from all individual participants included in the study.

According to the provider’s instructions, the tissue microarray was assembled using a commercially available manual tissue punch. The clinical tissue microarray contained relevant clinical information, such as sex, age, tumor–node–metastasis (TNM) stage, survival time, and programmed death ligand-1 (PD-L1) expression. The tissue microarray slide was stained with anti-HPSE antibody and anti-CD45 antibody. The expression levels of CD45 and PD-L1 were scored using a semiquantitative immunoreactivity scoring (IRS) system by three independent pathologists [14]. The percentage of positive cells was stratified using a system of six scores: 0 (no positive cells), 1 (<20% positive cells), 2 (21–40% positive cells), 3 (41–60% positive cells), 4 (61–80% positive cells), and 5 (>80% positive cells). The expression level of HPSE protein was quantified by using Image-Pro Plus 6.0 image analysis software. The “survival” package was used to perform Kaplan–Meier survival analysis with the log-rank test. The optimal cutoff value was ascertained by the surv_cutpoint function of the survminer R package.

### Statistical analyses

The statistical analyses were performed via R software (v 4.0.2) and GraphPad Prism 7.0 (San Diego, CA). Kaplan–Meier curves and log-rank tests were utilized to evaluate the survival data. A Spearman rank correlation was performed to evaluate the correlation between HPSE expression and clinicopathologic characteristics. The Wilcoxon test was mainly utilized for comparing two groups, and the Kruskal–Wallis test was used for two or more groups. A *p* value < 0.05 was considered to indicate statistical significance.

## Results

### Identification of DEGs between cancerous and adjacent esophageal tissues

**Fig. 1:**
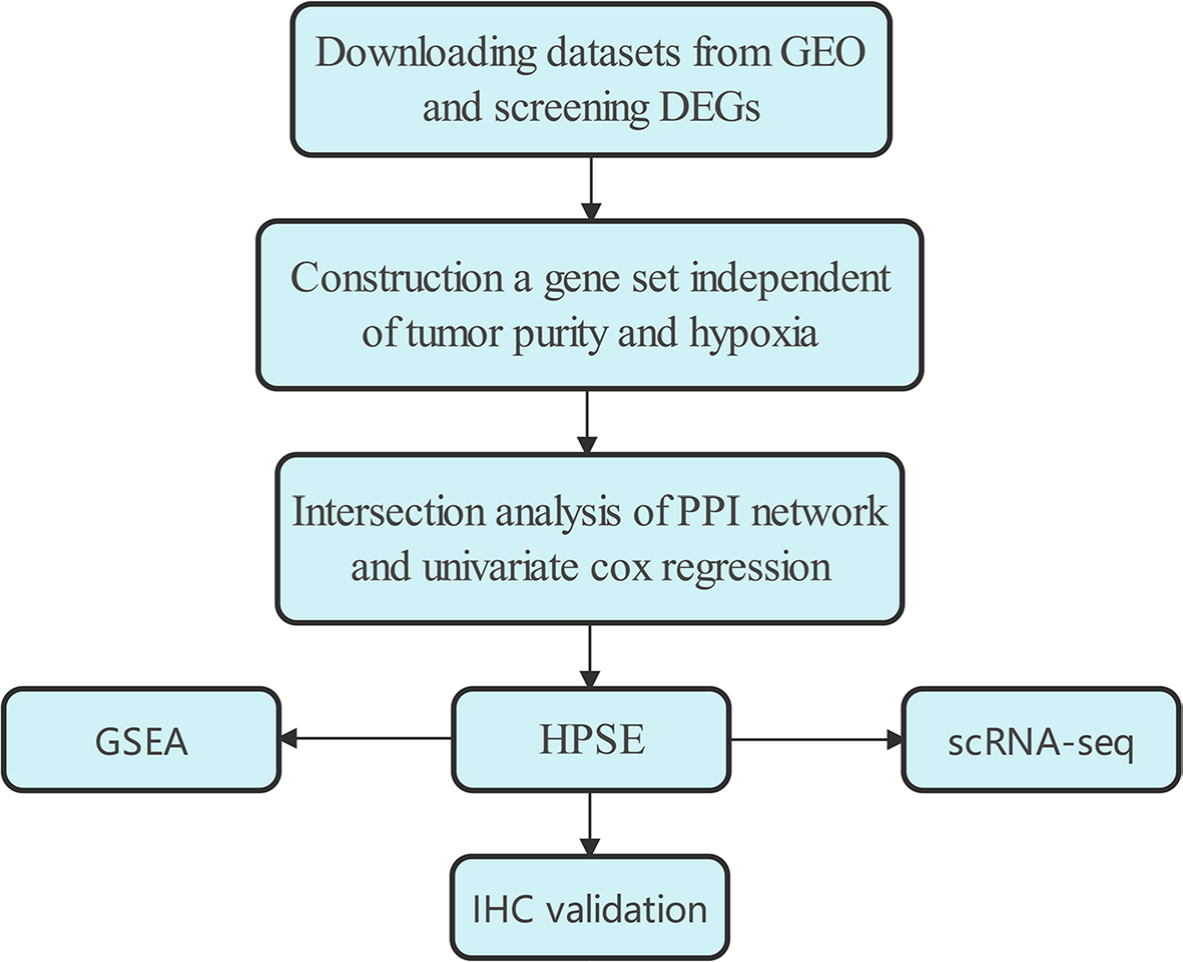
The flow chart of this study

**Fig. 2:**
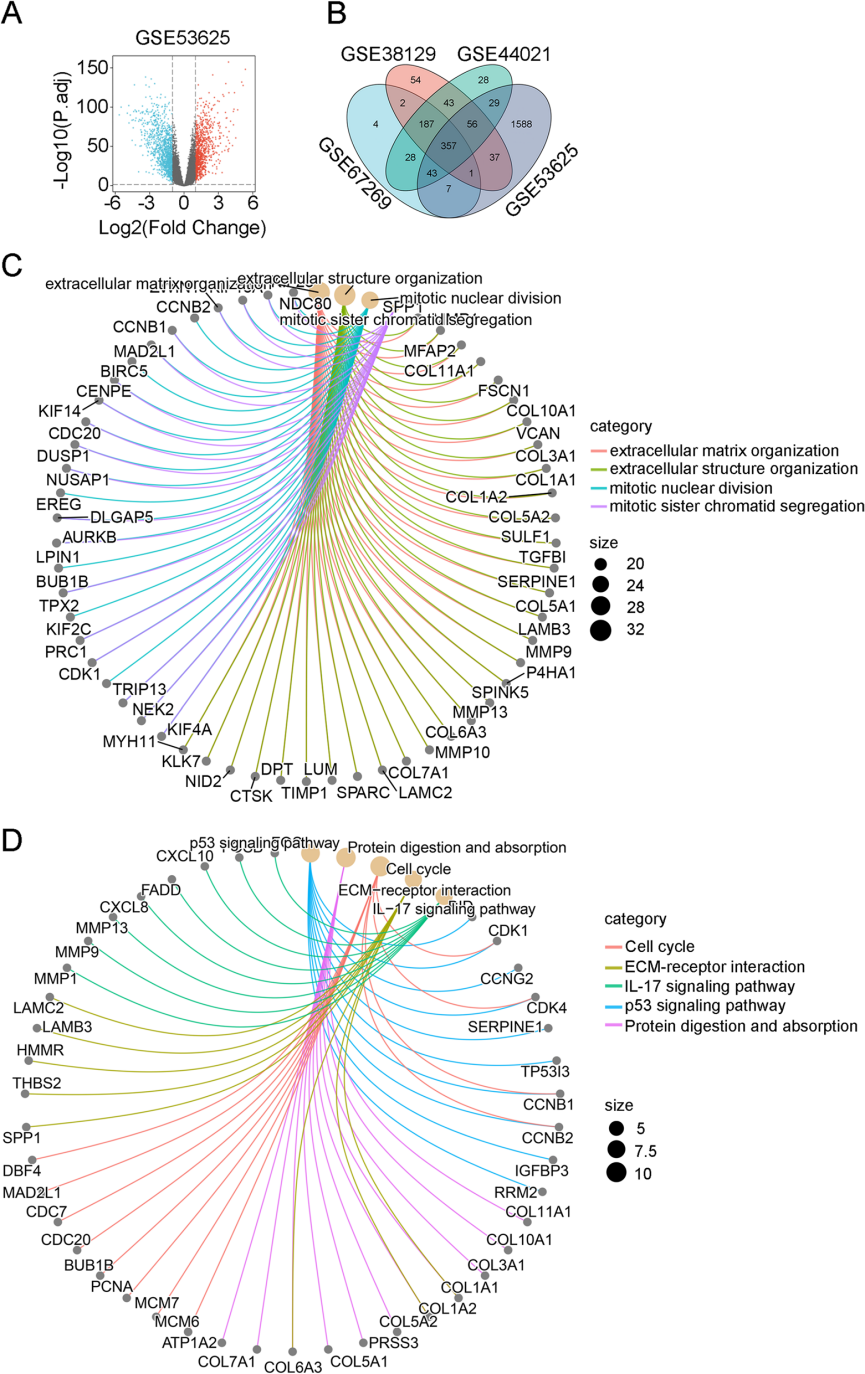
Identification of DEGs between cancerous and adjacent esophageal tissues. **A** Volcano map for the distribution of differentially expressed genes between tumor tissues and adjacent noncancerous tissues in esophageal squamous cell carcinoma samples. **B** Venn diagram showing the overlap between the four groups of differentially expressed genes. **C** GO analysis and **D** KEGG pathway enrichment analysis of differentially expressed genes

To compare gene expression between tumor and adjacent normal tissues in ESCC, we first converted GEO data probe matrixes into gene matrixes with R software. An overview of this study is shown in Fig. 1. Gene difference analysis revealed 628 DEGs in GSE67269, 736 DEGs in GSE38129, 771 DEGs in GSE44021, and 2118 DEGs in GSE53625. Volcano plots were used to depict the significant DEGs identified from the four GEO datasets (tumor versus normal tissues) (Fig. 2A, Fig. S1A-C). We further identified common differentially expressed genes (co-DEGs) in ESCC among the four microarray datasets (Fig. 2B); as shown in the Venn diagram, 357 common DEGs were identified from the four datasets. In ensuring analysis, we focused on the molecular functions of the 357 common significant DEGs. The results from Gene Ontology (GO) enrichment analysis indicated that these DEGs are related to collagen-containing extracellular matrix, extracellular structure organization, and enzyme inhibitor activity (Fig. 2C). Kyoto Encyclopedia of Genes and Genomes (KEGG) enrichment analysis indicated enrichment of the cell cycle pathway (Fig. 2D). Thus, we considered that these DEGs may be related to the metabolism of cellular components. We then analyzed these DEGs.

### Analysis of DEGs with expression not affected by tumor purity and hypoxia

**Fig. 3:**
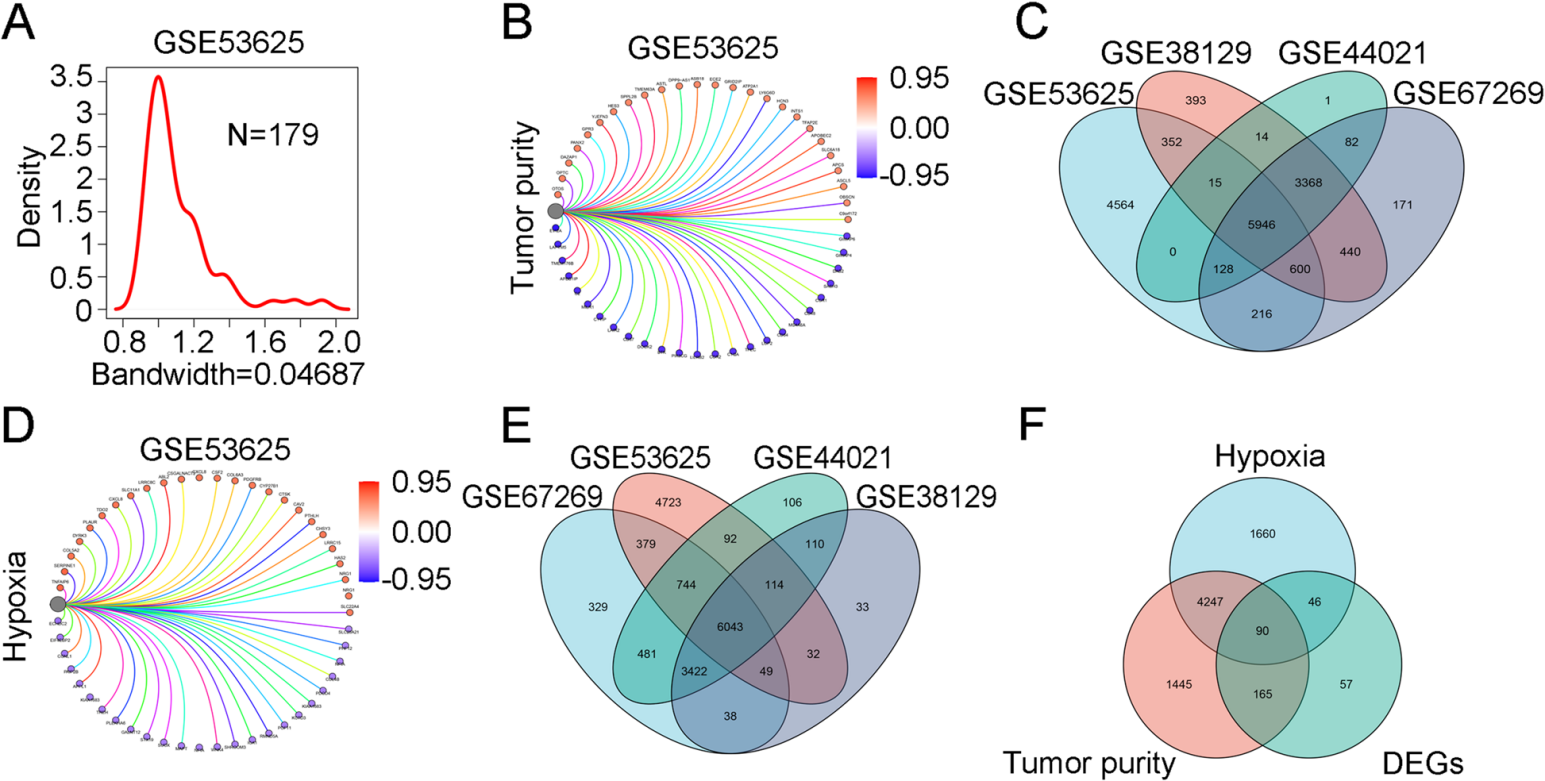
Analysis of DEGs with expression not affected by tumor purity and hypoxia. **A** Densogram representing the variance in tumor purity among tumor samples in GSE53625. **B** The network diagram presents correlations of gene expression and tumor purity, which were calculated using Spearman’s correlation analysis (|Spearman correlation coefficient| ≥ 0.4, *p value* ≤ 0.001). **C** The Venn diagram shows the overlapping genes among four groups of genes independent of tumor purity. **D** Genes correlated with hypoxia were identified in GSE53625 (|Spearman correlation coefficient| ≥ 0.4, *p value* ≤ 0.001). **E** The Venn diagram shows overlapping genes among four groups of genes independent of hypoxia. **F** DEGs independent of tumor purity and hypoxia in the three overlapping groups

Due to tumor heterogeneity and the complex tumor microenvironment, we tended to use tumor purity as a TME indicator. Given the complexity of the TME, prognostic genes unrelated to tumor purity and hypoxia were screened in our study. According to a previous analysis of the TME, we applied ESTIMATE algorithms to quantify the enrichment levels of immune and stromal cells in ESCC tumor tissues, and the tumor purity was calculated by this algorithm. The proportion of the main immune cells correlated inversely with tumor purity (Fig. S3A). As expected, tumor purity varied among the tumor samples (Fig. 3A, Fig. S2 A-C). To investigate the relationship of the tumor purity signature with expression of genes in each database, Spearman correlation was used to analyze the correlations. A total of 596 genes were associated with tumor purity in GSE53625 (Fig. 3B, Fig. S3B-D, |Spearman correlation coefficient| ≥0.4). By following the same method, the correlations of the other three groups were also calculate using the Spearman method. To mitigate the effect of tumor purity on tumor analysis, we selected genes unrelated to tumor purity from the four gene sets, which showed 5945 genes. (Fig. 3C). These genes were considered candidate genes for subsequent study.

Regional tumor hypoxia is one of the foremost features of solid tumors, including ESCC. To confirm the correlations between the DEGs and hypoxia-related genes, we first obtained 81 hypoxia-related genes as the hypoxia gene set from Molecular Signatures Database V7.2 and coexpression analysis was performed to confirm the hypoxia-related genes in each dataset via the gene set variation analysis (GSVA) method [15, 16] (Fig. 3D, Fig. S3E-G) |Spearman correlation coefficient| ≥0.4). We screened 6043 genes independent of hypoxia from the four gene datasets (Fig. 3E). Then, intersection analysis displayed by a Venn diagram showed 90 DEGs not influenced by tumor purity or hypoxia status (Fig. 3F). We further subjected these genes to functional enrichment analysis and GO and KEGG enrichment analyses indicated that most enriched pathways were related to epidermal development and cell cycle (Fig. S3H). These genes were used as a starting point for subsequent analysis.

### Intersection analysis of the PPI network and univariate cox regression

**Fig. 4:**
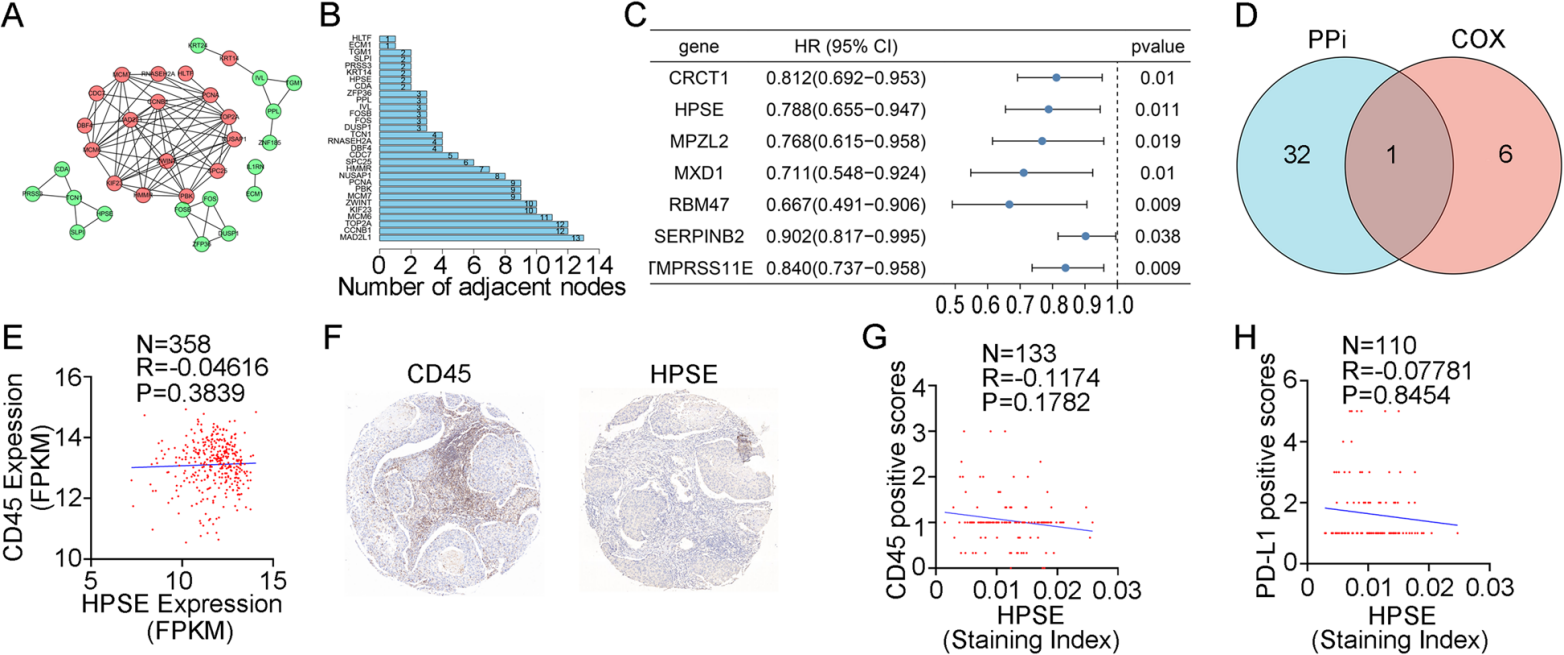
Intersection analysis of the PPI network and univariate Cox regression analysis. **A** An interaction network was built with nodes with interaction confidence values >0.90. **B** Genes are ordered by the number of nodes. **C** Univariate Cox regression analysis was performed on selected survival-related genes with a *p value* < 0.05. **D** Venn diagram displaying the intersection of the top 30 nodes in the PPI and the most significant factors in univariate Cox regression. **E** Relationship between the gene expression levels of CD45 and HPSE. **F** The expression patterns of HPSE and CD45 in ESCC tissues were assessed using immunohistochemistry. **G** The correlation between HPSE and CD45 in ESCC tissues was evaluated with Spearman rank correlation (*p value* = 0.1782). **H** The correlation between HPSE and PD-L1 in ESCC tissues was evaluated with a Spearman rank correlation (*p value* = 0.8454)

To investigate the expression of these genes at the protein level, we constructed a PPI network utilizing Cytoscape software based on the STRING database, and the network included 33 nodes and 84 edges (Fig. 4A). We next sorted the top 30 DEGs in bar plots according to the number of nodes (Fig. 4B). To identify DEGs associated with patient survival in ESCC, the clinicopathological characteristics of the 179 patients from the GSE53625 dataset were analyzed. Univariate Cox regression analysis of the DEGs was performed. The results revealed that 7 DEGs had prognostic value in ESCC (Fig. 4C). Then, we performed an intersection analysis of the top 30 core nodes in the PPI network and 7 significant factors in the Cox regression analysis (Fig. 4D). The only factor that overlapped was HPSE. Moreover, we investigated HPSE expression in 466 ESCC tissues and paired adjacent normal tissues from 4 cohorts and found it to be significantly lower in these cancer tissues than in the paired adjacent normal tissues (Fig. S4A-D). Thus, we found that low HPSE expression may be associated with a poor prognosis in ESCC patients.

Based on bioinformatics analysis, HPSE expression was not related to tumor purity or hypoxia. Thus, we used CD45 as a marker of immune cells and quantified the expression of CD45. At the gene expression level, no correlation was found between the expression of CD45 and HPSE (Fig. 4E). We performed IHC to further analyze the relationship between HPSE expression and CD45, which also showed no correlation (Fig. 4F, G). Hence, the expression observed at the protein level was consistent with that at the gene level. Then, we analyzed programmed death ligand-1 (PD-L1) expression in paraffin-embedded tumor tissue sections and found that it also did not correlate with HPSE expression in cancerous tissues (Fig. 4H). The conclusions above also reflected that the expression of HPSE was not influenced by tumor purity.

### HPSE is involved in multiple biological processes within ESCC tissues

**Fig. 5:**
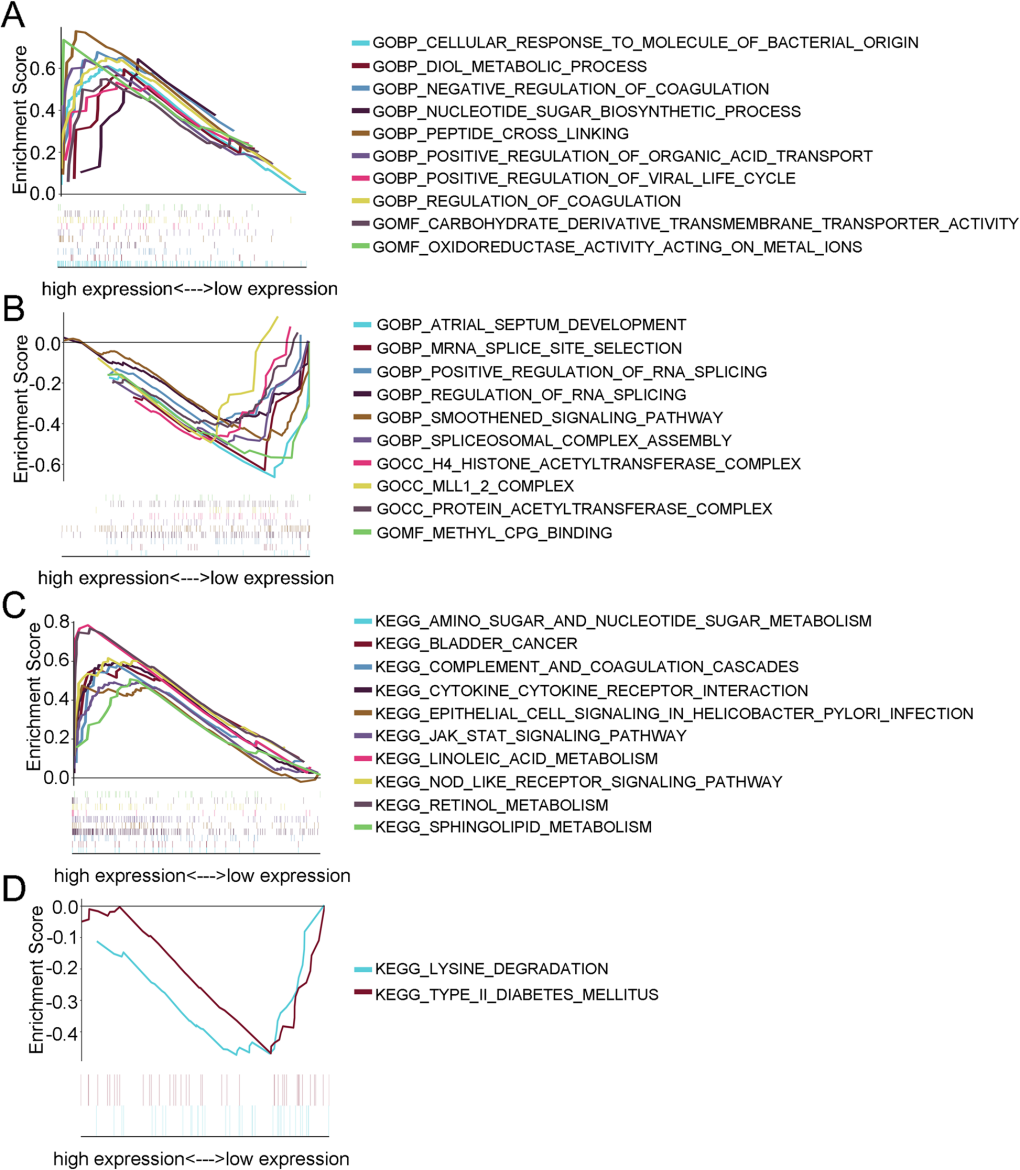
HPSE was involved in multiple biological processes in ESCC tissues. **A** The results of GSEA (GO terms) showed that the high HPSE expression group was enriched in oxidoreductase activity acting on metal ions, peptide cross linking, and the nucleotide sugar biosynthetic process. **B** The enriched GO pathways in samples with low HPSE expression. **C** The GSEA results (KEGG pathways) showed that samples with high HPSE expression were significantly enriched in cytokine–cytokine receptor interactions, the Jak-STAT signaling pathway, and retinol metabolism. **D** Enriched KEGG pathways in the low HPSE expression group

Given the above results, we sought to examine the potential biological processes of HPSE expression in ESCC. Gene set enrichment analysis (GSEA) was applied to compare high-expression and low-expression groups separated according to the median level of HPSE expression. The results showed that the enriched GO terms in the high HPSE group were mainly related to oxidoreductase activity acting on metal ions, peptide cross linking, and nucleotide sugar biosynthetic processes (Fig. 5A). For the HPSE low-expression group, biological processes, including atrial septum development, protein acetyltransferase complex, and regulation of RNA splicing, were enriched (Fig. 5B). Enrichment analysis of KEGG pathways was also conducted and indicated that high HPSE expression was related to cytokine–cytokine receptor interactions, the Jak-STAT signaling pathway, and retinol metabolism (Fig. 5C). We further observed significant enrichment of the lysine degradation pathway (Fig. 5D). On the other hand, the Reactome database was used to understand the steps of HPSE biological pathways [17]. The results indicated that HPSE enriched in many Reactome signaling pathways, which mainly were involved in HS-GAG degradation and heparan sulfate/heparin (HS-GAG) metabolism (Table S1). Additionally, we further explored differential expression of the HPSE functional partners predicted by the STRING database (https://www.string-db.org/) between ESCC tissues and para-carcinoma tissues (Fig. S5A) [13]. Glypican proteoglycan (GPC1 and GPC6), core membrane-anchored heparan sulfate proteoglycans, were upregulated in ESCC tissues compared with para-carcinoma tissues, whereas syndecan proteoglycans (SDC1, SDC2, and SDC4), transmembrane heparan sulfate proteoglycans, were downregulated (Fig. S5B). These results indicated that HPSE was involved in regulating the multi-step reaction of heparan sulfate glycosaminoglycans and proteoglycans, leading to extracellular matrix remodeling in ESCC. Taken together, these results suggest that HPSE is involved in multiple biological processes in ESCC.

### Single-cell RNA sequencing to explore HPSE expression in immune cells

**Fig. 6:**
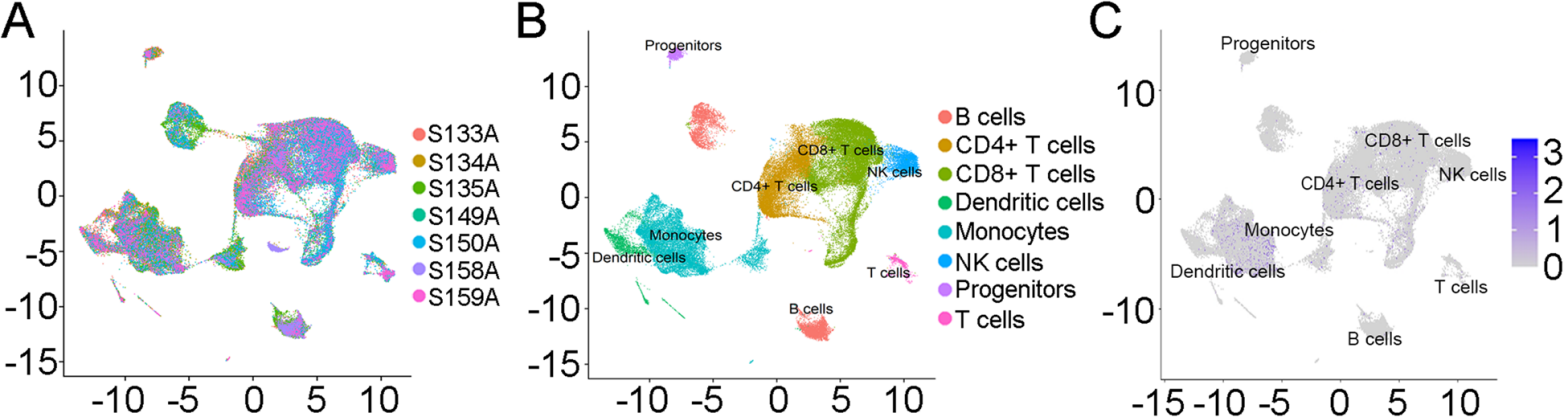
Single-cell RNA sequencing to explore HPSE expression in immune cells. **A** UMAP showed the visualization of 70,556 immune cell distributions in 7 esophageal squamous cancer tissues. **B** Based on the expression of known marker genes, the major types of tumor-infiltrating immune cells were identified, including B cells, T cells, CD4+ T cells, CD8+ T cells, dendritic cells, NK cells, monocytes, and progenitors. **C** Expression level of HPSE in each cluster

To validate the HPSE expression distribution in the tumor immune microenvironment, we further generated a transcriptional map of immune cells in human ESCC. Single-cell transcriptome data were obtained from dataset GSE160269 [18] in the GEO database. The raw sequencing data were filtered by removing data for low-quality cells. In total, 70,556 cells were identified from 7 tumor samples for further analysis (Fig. 6A). We next performed unsupervised clustering on all samples to identify distinguishable populations. Clustering and visualization were performed in R using the Seurat package. Based on the annotations of cells, we found the major types of tumor-infiltrating immune cells, including B cells, T cells, CD4+ T cells, CD8+ T cells, dendritic cells, NK cells, monocytes, and progenitors, to be present in ESCC (Fig. 6B), with HPSE predominantly located on monocytes (Fig. 6C). The results indicate that HPSE is likely to be mainly expressed on tumor cells and monocytes in the immune microenvironment.

### Low HPSE expression in ESCC tumor tissues is associated with poor prognosis

**Fig. 7:**
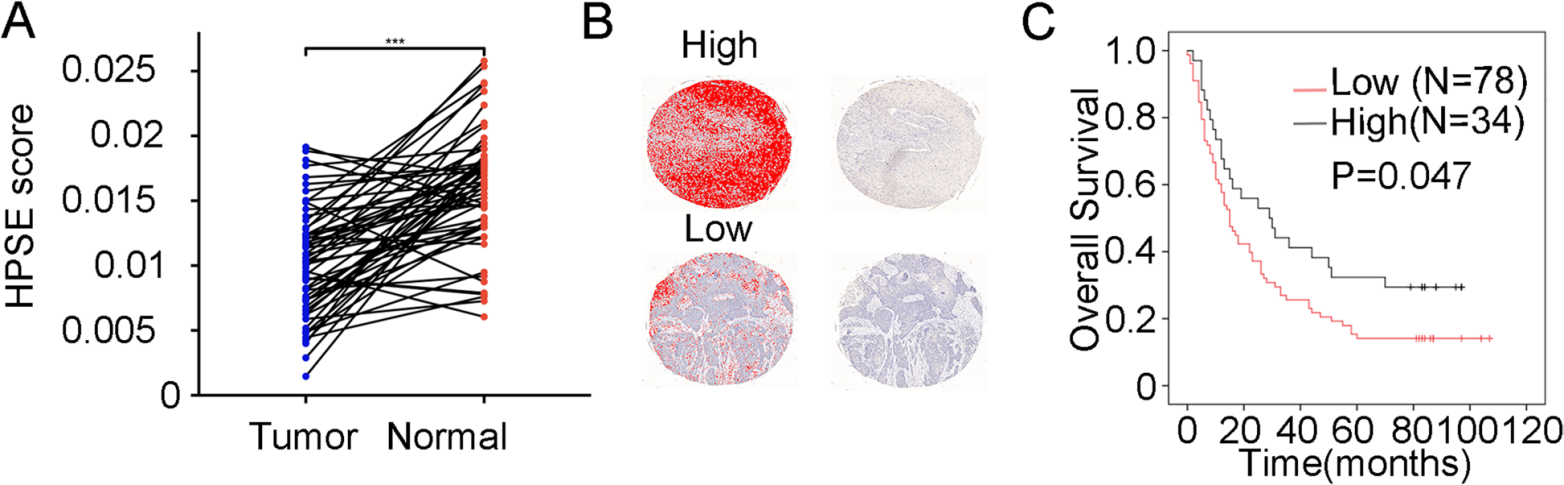
HPSE expression in ESCC tumor tissues is associated with a poor prognosis. **A** A paired *t* test was used to compare the differences in HPSE protein levels between ESCC and matched para-carcinoma tissues. **B** Representative IHC images for high and low HPSE expression. The expression level of HPSE protein was quantified by using Image-Pro Plus 6.0 image analysis software. **C** Kaplan–Meier curves showed that patients with low HPSE levels in cancerous gastric tissues had poor overall survival

Next, we applied immunohistochemistry analysis to validate the relative HPSE expression between ESCC tissues and paired adjacent normal tissues from 66 ESCC patients. HPSE expression was lower in ESCC tissues than para-carcinoma tissues, consistent with our genetic analysis (Fig. 7A). Subsequently, we further evaluated the relationship between HPSE expression and patient survival. The corresponding clinical data is listed in Supplemental Table S2. Analysis of ESCC tissues with complete clinicopathologic information consistently indicated that the expression of HPSE in cancerous tissues correlated negatively with clinicopathologic classifications N stage (*P* = 0.013, Table 1) and clinical stage (*P* = 0.024, Table 1). We divided patients into low- and high-expression HPSE groups by selecting optimal cutoff values with the survival R package. Kaplan–Meier curves showed that the overall survival time of patients with low HPSE expression in esophageal cancer tissues was shorter than that of those with high HPSE expression (log-rank test, *p* = 0.047) (Fig. 7B, C). Together, these results suggest that HPSE may be a potential prognostic marker for ESCC.

**Table 1:** Correlation between HPSE expression in cancerous tissues and clinicopathologic characteristics

Patient characteristic	*N*	Spearman *R*	*p* value
Gender	112	0.013	0.889
Male	83		
Female	29		
Age (years)	111	−0.041	0.673
≤67	57		
>67	54		
T classification	106	−0.132	0.177
T1	4		
T2	18		
T3	83		
T4	1		
N classification	112	−0.235	0.013*
N0	52		
N1	36		
N2	19		
N3	5		
Clinical stage	107	−0.218	0.024*
I	4		
II	48		
III	55		
IV	0		
Survival status	112	−0.120	0.208
Alive	21		
Death	91		

*p < 0.05

### Analysis of the effect of HPSE and clinical factors on the prognosis of ESCC

Previous data demonstrated a worse prognosis with a lower expression level of HPSE. To determine whether HPSE is a risk factor for prognosis in ESCC patients, we analyzed its expression and clinical significance in ESCC based on Kaplan–Meier and Cox proportional hazards regression models. By univariate Cox regression analysis, we found that low levels of HPSE expression in tumor tissues correlated with a significantly increased risk of cancer-related death in patients with ESCC (Table 2). Similarly, the univariate analysis showed sex, clinical stage, classification T, and classification N to be associated with prognosis (Table 2). However, age subgroup did not correlate with poor prognosis (Table 2). Subsequently, we performed multivariate Cox regression analysis of the five aforementioned factors. Clinical stage and sex remained poor prognostic factors in the multivariate Cox regression analysis of overall survival (Table 3). In particular, we found that compared with female patients, male patients had worse overall survival. Together, these data suggest that HPSE expression in ESCC tissues is associated with a poor prognosis and could serve as a risk factor to predict poor survival.

**Table 2:** Univariate Cox regression analysis of potential prognostic factors for esophageal squamous cancer patients in the tissue microarray

Patient characteristic	*N*	RR (95% CI)	*p* value
Gender	112		
Male	83	1	
Female	29	0.439 (0.258–0.747)	0.002**
Age (years)	111		
≤66	57	1	
>66	54	1.052 (0.695–1.593)	0.811
T classification	106		
T1+T2	22	1	
T3+T4	84	2.710 (1.490–4.931)	0.001**
N classification	112		
N0+N1	88	1	
N2+N3	24	2.749 (1.680–4.498)	0.000***
Clinical stage	107		
I–II	52	1	
III–IV	55	3.127 (1.970–4.961)	0.000***
HPSE expression	112		
Low	93	1	
High	19	0.525 (0.286–0.965)	0.038*

*RR* relative risk, *CI* confidence interval

*p < 0.05, **p < 0.01, ***p < 0.001

**Table 3:** Multivariate Cox regression analysis of potential prognostic factors for esophageal squamous cancer patients in the tissue microarray

Patient characteristic	*N*	RR (95% CI)	*p* value
Gender	106		
Male	79	1	
Female	27	0.543 (0.309–0.956)	0.034*
T classification	106		
T1+T2	22	1	
T3+T4	84	1.817 (0.937–3.525)	0.077
N classification	106		
N0+N1	83	1	
N2+N3	23	1.492 (0.850–2.618)	0.163
Clinical stage	106		
I–II	52	1	
III–IV	54	1.915 (1.088–3.370)	0.024*
HPSE expression	106		
Low	88	1	
High	18	0.727 (0.375–1.412)	0.347

*RR* relative risk, *CI* confidence interval

*p < 0.05, **p < 0.01, ***p < 0.001

## Discussion

Esophageal carcinoma is a common malignant tumor of the digestive tract with a poor prognosis [19]. ESCC is a common subtype in China that is characterized by unsatisfactory therapeutic efficacy as well as a poor prognosis [20]. Although TNM staging is widely used as the risk stratification system for ESCC patients [21], it is insufficient for predicting prognosis for some patients. In recent years, with the development of molecular biology and further understanding of the TME, several genes have been reported as prognostic factors in patients with ESCC [4, 22]. However, the TME is rather complex and significantly distinct in different tumor tissues [23, 24]. Thus, we applied tumor purity as the assessment criterion of the TME. Due to the differences in tumor purity and hypoxia across different tumor tissues, the replicability of previously established prognostic models based on bioinformatic analysis in ESCC is poor. It is also unclear how to use gene expression signatures to achieve better prognostic prediction in ESCC.

In our study, we attempted to explore a new gene signature closely related to the prognosis of ESCC through a series of bioinformatics analyses. The TME is important for tumor pathogenesis and can change the gene expression levels in tumor cells. Due to heterogeneity in gene expression, it is not convincing to identify cancer prognostic markers by analyzing the overall tumor tissues. On the other hand, hypoxia in the cancer microenvironment is a common feature of most malignant tumors and exerts an adverse effect in terms of tumor aggressiveness and patient prognosis [9, 25]. Correspondingly, hypoxia has a strong influence on gene expression in tumor tissues. Therefore, if genes selected are influenced by tumor purity, hypoxia, and other physical or biological influences, a prognostic model might have low validity. Thus, we screened new gene markers independent of tumor purity and hypoxia to construct a prognostic model in ESCC. By bioinformatics analysis, heparanase (HPSE) was identified as a prognostic biomarker in ESCC independent of tumor purity and hypoxia, a finding that was verified by IHC.

We performed a transcriptomic analysis of ESCC data in the GEO database. The ESTIMATE algorithm was used to calculate tumor purity according to immune and stromal scores. From the correlation analysis, we selected a group of genes that had no correlation with tumor purity. Next, we constructed a gene set related to hypoxia based on pathway analysis by GSVA. We focused on the intersection of gene sets, which included DEGs not influenced by tumor purity and hypoxia. By means of gene enrichment analysis, we explored the biological processes of the co-expressed genes. Interestingly, functional analysis revealed that the intersecting genes were associated with epidermal development and the cell cycle. Subsequently, we constructed a PPI network and performed univariate Cox regression analysis. The results indicated that HPSE may predict the prognosis of ESCC independent of the TME and tumor purity. We explored the biological function of HPSE, and GSEA results showed that HPSE correlated with oxidoreductase activity acting on metal ions, septum development, growth and signal transduction, and lysine degradation pathways. These results indicate that HPSE is involved in multiple biological processes in ESCC. However, the pathways enriched in samples with high HPSE expression were not associated with tumor purity or hypoxia. We also used the Reactome database [17] and PPI network to understand the steps of HPSE biological pathways. The results indicated that HPSE is involved in regulating the multi-step reaction of heparan sulfate glycosaminoglycans and proteoglycans, leading to extracellular matrix remodeling in ESCC. We also analyzed the expression of HPSE in immune cells by single-cell sequencing and observed HPSE expression in both tumor tissues and para-cancerous tissue-resident immune cells.

HPSE is an endo-β-D-glucuronidase capable of cleaving heparan sulfate side chains that are liberating such heparan sulfate binding proteins, as well as potentially contributing to extracellular matrix (ECM) degradation [26, 27]. As a degrading enzyme, HPSE is involved in biological and pathological processes, including tissue repair, inflammation, tumor angiogenesis, invasion, and metastasis [28]. Some studies have reported that upregulated HPSE correlates with poor prognosis in myeloma, colon, breast, and prostate carcinoma [29, 30]. However, conflicting results were reported in gastric carcinomas, head and neck carcinomas [31], and lung cancers [32]. It has been reported that HPSE expression is notably reduced in hepatocellular carcinoma (HCC) tissues compared with non-tumor liver tissues and is significantly associated with poor outcomes [33]. In our research, we found that HPSE expression was downregulated in ESCC tissues and was associated with poor prognosis and lower rates of survival. The inconsistent conclusions from different reports on HPSE in different types of tumors might result from the different protein subcellular locations, expression levels, and activities of HPSE. In addition, the heparan sulfate (HS) side chains of heparan sulfate proteoglycans (HSPGs) can bind multiple growth factors, chemokines, cytokines, and enzymes in the ECM and cell surface [34–36]. HPSE release HS-bound growth factors, such as basic fibroblast growth factor (bFGF), by cleaving HSPG side chains in hepatocellular carcinoma (HCC) [37, 38]. bFGF may promote tumor progression by enhancing endothelial cell and tumor cell proliferation [39]. HS-bound growth factors exhibited inhibitory effects on proliferation and signaling activation of tumor cells, such as melanoma and HCC. Thus, low HPSE expression might enhance the ability of growth factors to bind to esophageal tumor cells and confer worse prognosis. Regardless, the detailed mechanism of its regulation requires further study.

To verify these results of bioinformatic analysis, we further analyzed the expression in ESCC samples of a tissue microarray. As expected, we found that the expression of HPSE was lower in these cancer tissues than in the paired adjacent normal tissues and was significantly associated with survival. We also analyzed the correlation between CD45 and PD-L1 immune indexes and HPSE expression and found that HPSE expression was not correlated with that of PD-L1. The conclusions also reflected that the expression of HPSE was not influenced by tumor purity. Moreover, we used univariate Cox proportional hazard analysis to evaluate risk factors for clinical prognosis, and the results indicated that clinical stage, T classification, and M classification were associated with prognosis. It was also shown that HPSE expression was correlated with metastasis in ESCC. We also found that sex was a risk factor for ESCC, with patients having worse overall survival [40]. As previously reported, female patients had better survival outcomes than male patients in ESCC, consistent with our results [41, 42]. Preclinical studies have demonstrated that estrogens may inhibit growth of squamous tumor cells. Thus, the relatively better survival in female compared with male patients with ESCC may be at least partly explained by the influence of sex hormones. However, efforts should be made to investigate the underlying biological mechanism. Based on multivariate Cox regression analysis, the current study showed that HPSE expression correlated significantly with clinical stage and sex in patients with ESCC. Although HPSE has been proposed as a prognostic marker in ESCC, it is unclear whether and how it plays a role in tumor metastasis.

In summary, HPSE is a potential prognostic marker independent of tumor purity and hypoxia influences. Our findings suggest that HPSE is a novel prognostic marker for patients with ESCC. Nevertheless, the mechanisms by which HPSE regulates ESCC carcinogenesis and metastasis have not yet been elucidated and warrant further investigation.

## Conclusion

In conclusion, the purpose of the current study was to determine a better way to predict the prognosis of ESCC. We identified and verified that the expression of HPSE was independent of tumor purity and hypoxia and clearly correlated with the metastasis and prognosis of ESCC. Thus, our study will be helpful in ESCC patient evaluation and has significant clinical relevance.

## Supplementary Information

**Additional file 1: Figure S1.** (A-C) Volcano plots of differentially expressed genes in GSE38129, GSE44021 and GSE67269.

**Additional file 2: Figure S2.** (A-C) Densogram representing the variance of tumor purity among tumor samples in GSE38129, GSE67269 and GSE44021.

**Additional file 3: Figure S3.** (A) Immune cell infiltrates were estimated by ssGSEA algorithm. (B-D) The network diagram presents correlations of gene expression and tumor purity in GSE38129, GSE44021 and GSE67269. ssGSEA, single-sample gene set enrichment analysis. (E-G) The correlation of gene expression and hypoxia was identified in GSE38129, GSE44021 and GSE67269. (D) GO analysis and KEGG enrichment analysis of 90 DEGs (*p-value*<0.05 indicated significant enrichment).

**Additional file 4: Figure S4.** (A-D) The differences in HPSE mRNA expression between ESCC tissues and adjacent normal tissues were evaluated by the Wilcoxon matched-pairs signed rank test in GSE53625, GSE67269, GSE44021 and GSE38129.

**Additional file 5: Figure S5.** (A) PPI network construction was used for protein interaction analysis. The Search Tool for the Retrieval of Interacting Genes/Proteins (STRING) database was used to evaluate protein–protein interaction (PPI) network information (https://www.string-db.org/). (B) The expression of interacting genes was compared between ESCC tissues and adjacent normal tissues.

**Additional file 6: Table S1.** Results of the Reactome pathway analysis.

**Additional file 7: Table S2.** Detailed characteristics of patients in the tissue microarray.

## Data Availability

All data generated or analyzed during this study are included in this published article and its supplementary information files.

## Abbreviations

TME: Tumor microenvironment
EAC: Esophageal adenocarcinoma
ESCC: Esophageal squamous cell carcinoma
DEGs: Differentially expressed genes
PPI: Protein–protein interaction
IHC: Immunohistochemistry
GSEA: Gene set enrichment analysis
HPSE: Heparanase
GEO: Gene Expression Omnibus database
GO: Gene Ontology
KEGG: The Kyoto Encyclopedia of Genes and Genomes
GSVA: Gene set variation analysis
PD-L1: Programmed death ligand-1
PCA: Principal component analysis
UMAP: Uniform manifold approximation and projection
TNM: Tumor–node–metastasis
IRS: Immunoreactivity scoring
bFGF: Basic fibroblast growth factor
HSPGs: Heparan sulfate proteoglycans
HS: Heparan sulfate
ECM: Extracellular matrix
