# Supplementary figures and images for: Heparanase is a prognostic biomarker independent of tumor purity and hypoxia based on bioinformatics and immunohistochemistry analysis of esophageal squamous cell carcinoma
Source: World J Surg Oncol. 2022 Jul 16;20:236. doi: 10.1186/s12957-022-02698-9 (PMC9288057; doi:10.1186/s12957-022-02698-9)

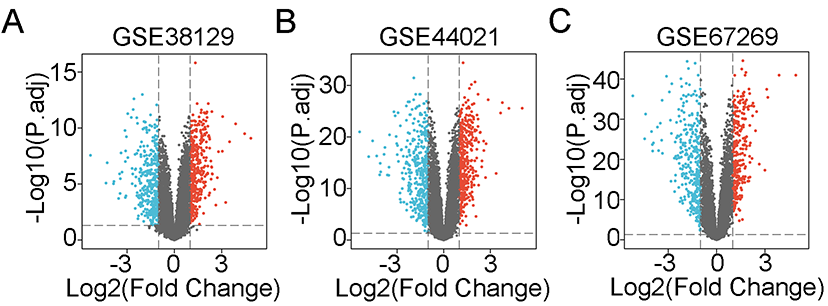

Supplement: Supplementary file 1 — Additional file 1: Figure S1. (A-C) Volcano plots of differentially expressed genes in GSE38129, GSE44021 and GSE67269. [file 12957_2022_2698_MOESM1_ESM.tif]

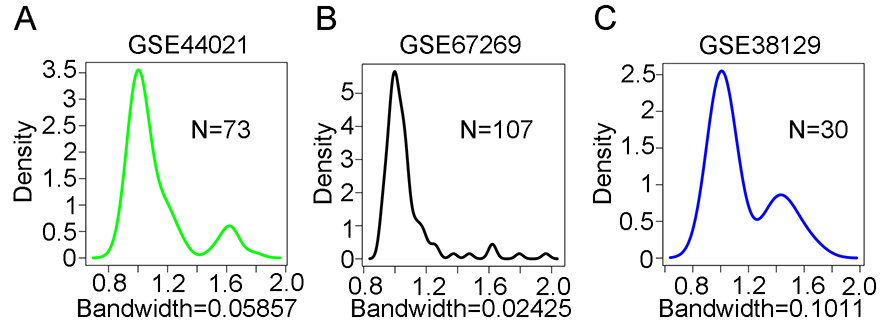

Supplement: Supplementary file 2 — Additional file 2: Figure S2. (A-C) Densogram representing the variance of tumor purity among tumor samples in GSE38129, GSE67269 and GSE44021. [file 12957_2022_2698_MOESM2_ESM.tif]

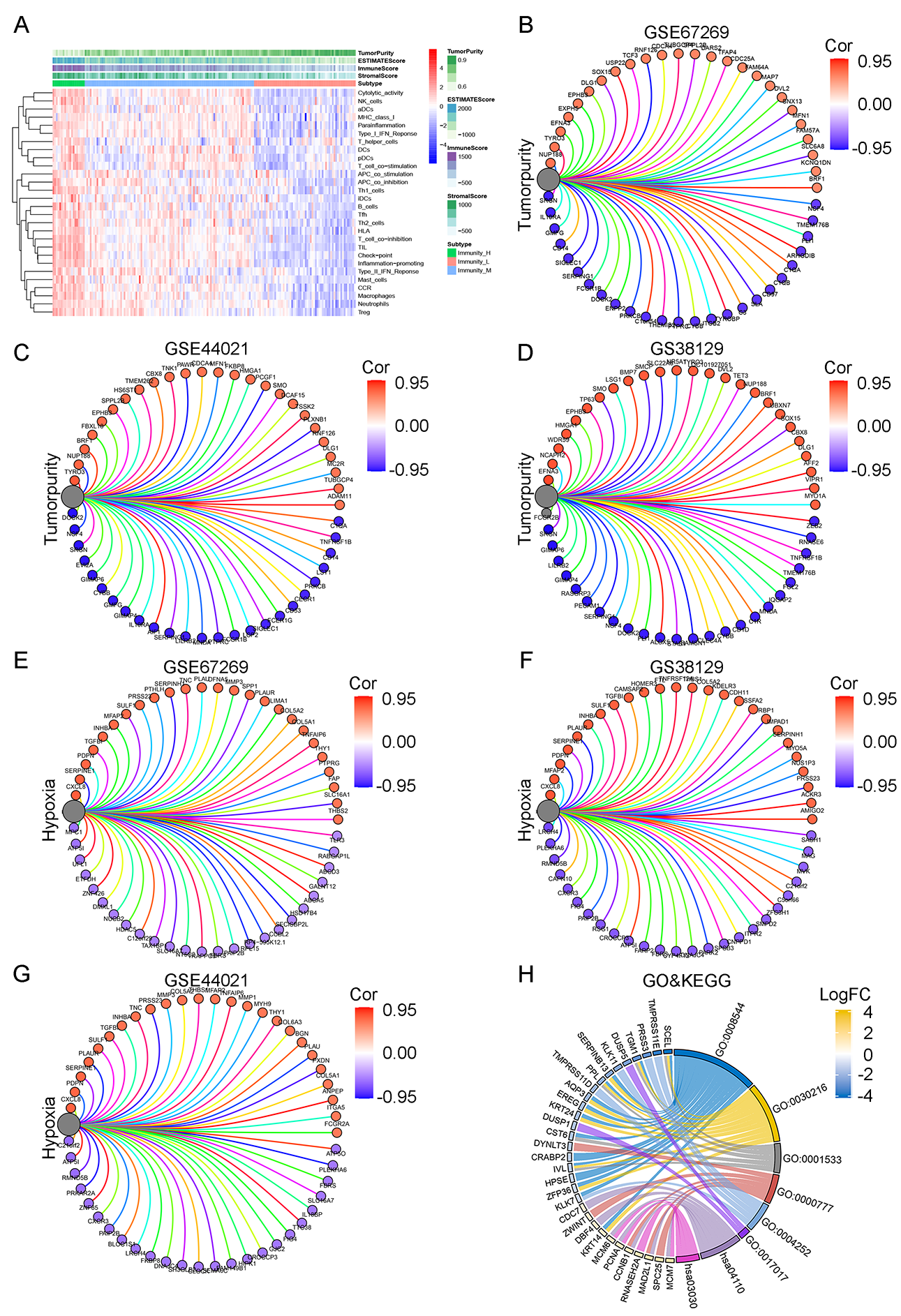

Supplement: Supplementary file 3 — Additional file 3: Figure S3. (A) Immune cell infiltrates were estimated by ssGSEA algorithm. (B-D) The network diagram presents correlations of gene expression and tumor purity in GSE38129, GSE44021 and GSE67269. ssGSEA, single-sample gene set enrichment analysis. (E-G) The correlation of gene expression and hypoxia was identified in GSE38129, GSE44021 and GSE67269. (D) GO analysis and KEGG enrichment analysis of 90 DEGs (p-value<0.05 indicated significant enrichment). [file 12957_2022_2698_MOESM3_ESM.tif]

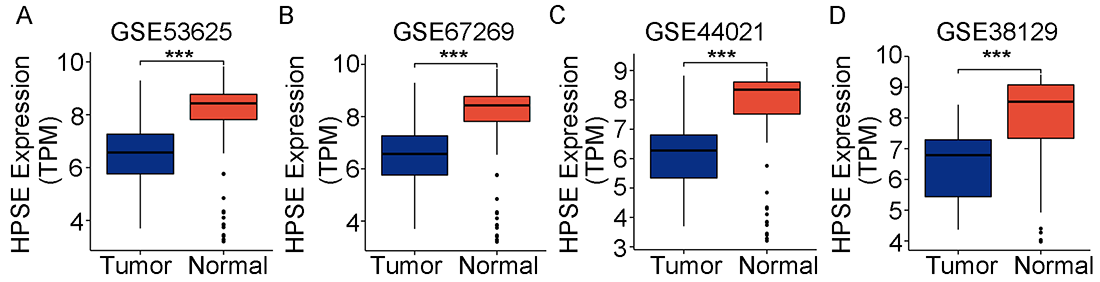

Supplement: Supplementary file 4 — Additional file 4: Figure S4. (A-D) The differences in HPSE mRNA expression between ESCC tissues and adjacent normal tissues were evaluated by the Wilcoxon matched-pairs signed rank test in GSE53625, GSE67269, GSE44021 and GSE38129. [file 12957_2022_2698_MOESM4_ESM.tif]

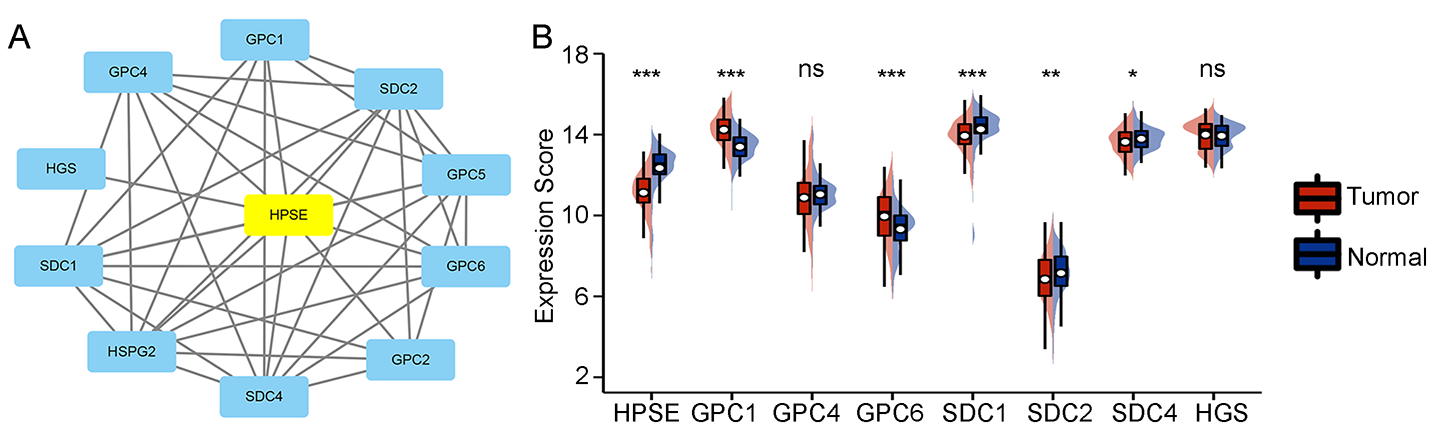

Supplement: Supplementary file 5 — Additional file 5: Figure S5. (A) PPI network construction was used for protein interaction analysis. The Search Tool for the Retrieval of Interacting Genes/Proteins (STRING) database was used to evaluate protein–protein interaction (PPI) network information (https://www.string-db.org/). (B) The expression of interacting genes was compared between ESCC tissues and adjacent normal tissues. [file 12957_2022_2698_MOESM5_ESM.tif]
