# Supplementary material for: Heparanase is a prognostic biomarker independent of tumor purity and hypoxia based on bioinformatics and immunohistochemistry analysis of esophageal squamous cell carcinoma
Source: World J Surg Oncol. 2022 Jul 16;20:236. doi: 10.1186/s12957-022-02698-9 (PMC9288057; doi:10.1186/s12957-022-02698-9)
Supplement: Supplementary file 6 — Additional file 6: Table S1. Results of the Reactome pathway analysis. [file 12957_2022_2698_MOESM6_ESM.doc]

| Pathway name | Entities | | |
| --- | --- | --- | --- |
|  | ratio | *P-value* | FDR^*^ |
| HS-GAG degradation | 0.001 | 0.001 | 0.01 |
| Heparan sulfate/heparin (HS-GAG) metabolism | 0,003 | 0.003 | 0.013 |
| Glycosaminoglycan metabolism | 0.008 | 0.008 | 0.016 |
| Metabolism of carbohydrates | 0.024 | 0.024 | 0.026 |
| Neutrophil degranulation | 0.026 | 0.026 | 0.026 |
| Innate Immune System | 0.157 | 0.157 | 0.157 |
| Metabolism | 0.233 | 0.233 | 0.233 |
| Immune System | 0.299 | 0.299 | 0.299 |

**Table S1** The following table shows the 8 most relevant pathways sorted by *P-value*

*False Discovery Rate
